# 

**Published:** 1895-06-22

**Authors:** 


					Titid V'l!' 1G, Supplied under Royal Warrant to Her Majesty the Queen. June 22nd, 1895.
4T I ? ?? THE KING OF NATURAL TABLE WATERS. "1 ? ?>>
uopinia *f:M h 6 tJoFanni^
?/???|
< Cl/(scary
'VI
No. 456. Vol xvin. WITH EXTRA NURSING SUPPLEMENT. 'S. ?pFE*rcE.
a ?           * Per Annum, 10g. 6d., post free.
^-TTTRDAV Tttwv 29-NTTt 1895 [Registered at the General Post Offloe as a Newspaper for Monthly Parts, 6d.; post free,8d.
' " ui^ ' ? transmission in the United Kingdom and Abroad.] Ditto per Annum, 8 a. 6d., post free.

				

